# Locking plate osteosynthesis of scaphoid nonunion with severe bone defects: a case series

**DOI:** 10.1038/s41598-022-12305-2

**Published:** 2022-05-19

**Authors:** Kristian Welle, Stefan Taeger, Christian Prangenberg, Roslind Karolina Hackenberg, Jan-Dirk Kieback, Koroush Kabir

**Affiliations:** 1 Department of Orthopedics and Trauma Surgery, University Hospital of Bonn, Venusberg-Campus 1, 53127 Bonn, Germany; 2 Department of Orthopedics and Trauma Surgery, Hopitaux Robert Schuman, 9, rue Edward Steichen, 2540 Luxembourg, Luxembourg

**Keywords:** Fracture repair, Musculoskeletal system, Bone, Trauma

## Abstract

The treatment of scaphoid nonunion can be challenging with increasing defect size. We evaluated the outcome of scaphoid nonunion with a substantial bone defect treated with a multidirectional locking plate combined with cancellous bone grafting only. Thirteen patients with significant osseous defects measuring 6 mm or more suffering from primary nonunion without treatment (n = 6) or recalcitrant nonunion following Herbert screw osteosynthesis (n = 7) were treated and reviewed retrospectively. The stabilization was performed after debridement, autologous cancellous bone grafting and volar locking plate osteosynthesis. After a mean follow-up period of 12 months, 12 of the 13 patients achieved successful unions with a free range of motion and complete remittance of pain in everyday activity. The mean scapholunate angle decreased from 59.7° ± 11 to 43.9° ± 5 (effect strength d:1.7, p < 0.00001), scaphoid humpback deformity angle from 58.9° ± 8 to 45.1° ± 8 (d:1.8, p < 0.0001), whereas strength of the injured hand increased from 36.4 kg ± 10 to 42.4 kg ± 9 (d:1.4, p < 0.0001). One nonunion persisted without fragment dislocation receiving revision after 1 year. Thus, locking plate osteosynthesis with cancellous bone grafting is a valid alternative in scaphoid nonunions with extensive bone defects. Additionally, stable retention of the fragments prevents dislocation even in delayed or persistent nonunion. Further prospective studies are required to confirm these findings.

## Introduction

The fracture of the scaphoid is an injury prevalent in young patients^1^. In the case of early diagnosis and therapy, the results are satisfactory in the sense of healing^2^.

However, in a high number of patients, minor sustained trauma and marginal complaints lead to a delayed consultation. In these cases, a high proportion of primary nonunions is found due to late diagnosis and missed treatment. Since the scaphoid fracture accounts for approximately 5% of all fractures of the body, the occurrence of primary and secondary nonunions is a frequently encountered problem^3,4^. Even after surgical treatment of the nonunion, healing rates of only 80–90% are achieved and yet lower in case of avascular necrosis^5–9^.

The standard surgical treatment of a nonunion is an osteosynthesis of the scaphoid via Herbert screw or K-wire. The meta-analysis of Pinder et al.^6^ showed no difference in the consolidation rate between these two procedures. Additionally, this study found no significant difference in the consolidation rate between the use of vascularised and non-vascularised bone grafts, being 92% and 88%, respectively.

For several years, now, an anatomic volar locking plate for the scaphoid has been available as an alternative approach^10^. First assessments of these locking plates in the treatment of scaphoid fractures showed high union rates. Even after unsuccessful nonunion fixation with a Herbert screw, fusion rates from 89 to 100% were achieved^10–15^.

In contrast to the Herbert screw fixation, in our opinion, the advantage of the plate is to be found especially in the treatment of unstable nonunions with a dorsal intercalated segment instability (DISI) and in revision surgeries. When encountering a large bone defect after resection of the nonunion, the standard procedure is wedge grafting^16^. However, although this technique can provide highly successful union rates, it is technically challenging due to the need to shape the wedge graft accurately and to place the screw in such a way as to fixate 3 bone fragments. This procedure can become even more challenging in revision surgery. After removal of an intraosseous screw, the bone may be hollowed out, precluding a firm hold of a new screw. In these cases, it might be necessary to resort to an alternative form of bone stabilization, which can be provided by the volar locking plate.

Although the locking plate has been available for several years, there have only been few publications on surgical results and overall outcome. In 2016, Dodds and Halim conducted a retrospective study evaluating patients’ outcome after plate osteosynthesis with a scaphoid defect zone of more than 7 mm^17^. However, they did not use the locking screw mechanism but used the plate as a regular volar buttress plate. All patients were treated with an additional vascularized bone graft and postoperative ultrasound bone stimulation (exogen). The study showed a consolidation of the nonunion in 8 of 9 cases.

In 1988, Stark et al. showed that it is sufficient to fill the bone defect with cancellous bone in combination with a stable osteosynthesis to achieve an excellent consolidation rate of 97%^18^. Compared to vascularized bone grafts and external ultrasound bone stimulation, cancellous bone grafting of the scaphoid, either from the iliac crest or the distal radius, presents a simple procedure alongside with a reduction in operation time while sparing costs for bone stimulation.

Furthermore, a plate osteosynthesis provides stability superior to a single compression screw osteosynthesis, as shown in the cadaveric study by Mandaleson et al.^19^. The utilization of the locking plate mechanism of the volar plate, should, therefore, provide a very stable osteosynthesis with good fragment retention.

Our study aimed to evaluate if patients with large bone defects of the scaphoid in patients undergoing revision surgery after scaphoid nonunion a locking-plate osteosynthesis with isolated cancellous bone graft can achieve a union.

## Materials and methods

In this study, all patients with primary and secondary defect pseudarthrosis of the scaphoid, who received surgical treatment using a volar, multidirectional locking plate osteosynthesis between 2012 and 2016, were included. All patients presenting with a recalcitrant scaphoid nonunion received a thin layer computed tomography (CT) scan in addition to X-rays. Before the surgical intervention, the defect size, as well as the size and dislocation of the proximal fragment, were evaluated.

In addition, avascular necrosis (AVN) of the proximal pole was excluded by preoperative MRI and findings of bleeding upon puncture^20^.

Inclusion criteria for locking plate osteosynthesis were

- Primary or secondary defect zone
- Defect size of 6 mm or more measured intraoperatively after resection of sclerotic bone and preliminary restoration of the scaphoid geometry.

All other cases were treated with compression screw osteosynthesis and excluded from this study. Patients with a necrosis of the proximal pole or a very proximal pole fracture, in which anchoring at least 2 screws in the proximal fragment was not possible, were excluded from the study, too.

This study was approved by our Institutional Review Board of the University Hospital Bonn, Germany (# 406/17) and performed in accordance with the ethical standards as laid down in the 1964 Declaration of Helsinki and its later amendments. Written informed consent was obtained from all participants or, if participants are under 18, from a parent and/or legal guardian.

### Operative treatment

**Figure 1:**
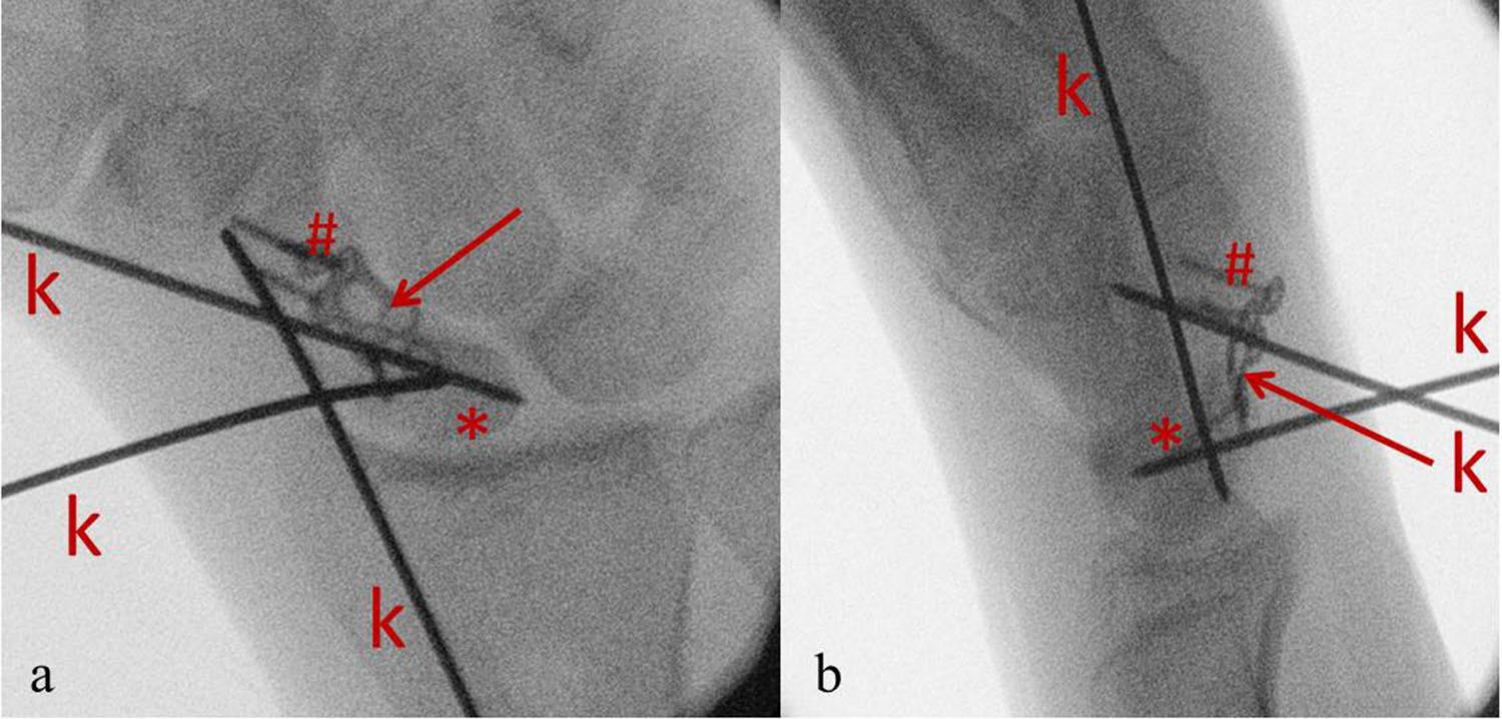
Intraoperative X-ray anteroposterior (**a**) and lateral (**b**) of scaphoid reposition and plate positioning. Using 2 K-wires (k) as joysticks, the proximal (*) and distal fragment (#) of the scaphoid are aligned and fixed with a third. The scaphoid plate (arrow) bridges the defect between the fragments via inserted angle-stable screws.

In all patients, an open pseudarthrosis resection was performed using a volar approach. In the course of the operation, the nonunion site and the sclerosis were completely resected. The lunate was repositioned and fixated with a k-wire under fluoroscopy. After repositioning the scaphoid, usually achieved by forcefully extending the wrist, the locking plate was positioned under fluoroscopic control (Fig. 1).

In all cases, a volar, multidirectional locking plate was used (APTUS^®^ Scaphoid Plate, Medartis AG, Basel, Switzerland). In case of an unstable situation, the distal fragment of the scaphoid was temporarily transfixed with a k-wire. Due to the standardized intrascaphoid angle predetermined by the plate, no adaptation of the plate angle was required. Only in 2 cases, it became necessary to shorten the plate to avoid an impingement due to the particularly small size of the proximal fragments. After placement of the plate and temporary fixation with k-wires, the wrist was moved under fluoroscopy to check for plate impingement in the end positions. Due to the multidirectional locking mechanism, we placed 6 locking screws in total, 3 screws on the proximal side and 3 screws on the distal side in all cases except for the two shortened plates where only 2 locking screws were placed in the proximal fragment.

The defect was augmented in all cases using autologous cancellous bone grafting from the iliac crest or distal radius and compressed by way of impaction grafting. For the extraction of the cancellous bone, a separate incision over the dorsal radius or the pelvic crest was necessary.

### Postoperative procedure

Concluding the operation, the forearm was immobilized under thumb enclosure for 6 weeks. No bone stimulation with ultrasound was applied. After 6 weeks, we began mobilization with the full range of motion under pain adapted weight-bearing. Full weight-bearing was reached in all patients after 3 months at the latest.

### Follow-up

**Figure 2:**
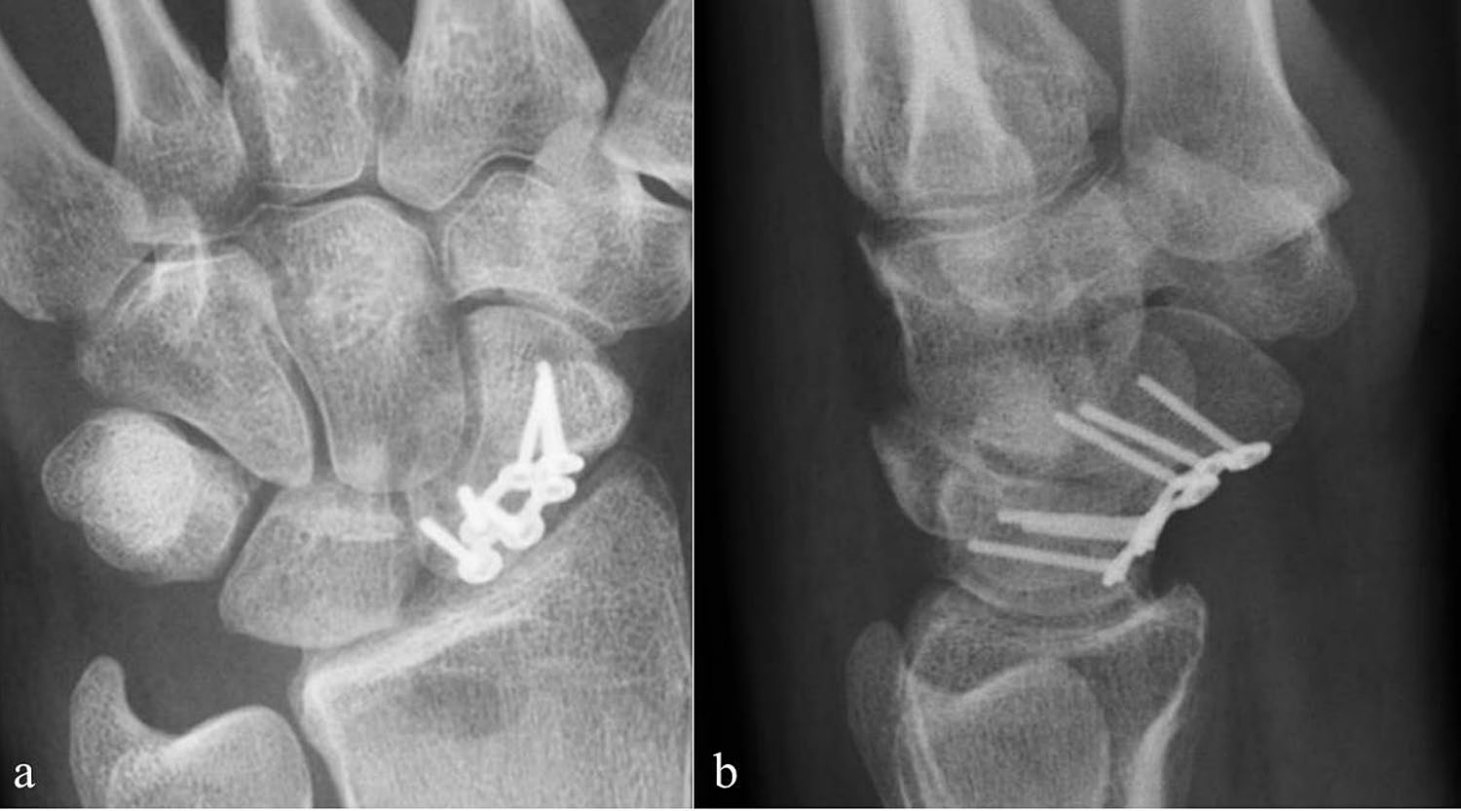
Control X-ray anteroposterior (**a**) and lateral (**b**) with the consolidation of the right scaphoid 6 months postoperative.

Patients were examined 2 and 6 weeks as well as 3, 6 and 12 months after surgery. X-rays were taken 6 weeks and 6 months after surgery (Fig. 2), CT scans were done 3 months following surgery.

In the final follow-up, the grip strength and the range of motion were measured and compared to the non-injured side. The pain level was identified using a visual analogue scale (VAS), and everyday functionality was evaluated by the Quick-DASH score^21^. Also, the patients were questioned about the time elapsed until they could return to work and return to preoperative levels of sports activities. The preoperative and postoperative scapholunate angles, the carpal height, and the intrascaphoid angle were measured via X-rays^22,23^ in the existing imaging to evaluate the preoperative dislocation and postoperative reduction. The scapholunate angle was measured using the tangent/line through the proximal and distal scaphoid poles and the line through the center of the medullar radial canal in the lateral wrist X-ray. The carpal height was measured from the proximal to the distal pole in the anteroposterior X-ray. The intrascaphoid angle was measured using the line through the center of the proximal und distal fragment in the lateral X-ray of the wrist. A scapholunate angle of > 60° was defined as pathologic. All measurements were carried out blinded by 2 independent senior surgeons. In case of a deviation, the mean value of both measurements was calculated.

### Statistics

The data of the patients were statistically analyzed with BiAS (H. Ackermann, Frankfurt, Germany). Mean values, standard deviations, confidence intervals 95% (CI: 95%), medians and quarters 2 and 3 were calculated.

The simple t-test was used for the comparisons of parametric values (before and after the surgery). The Welsh test evaluated comparisons between two groups of parametric values. In addition, Cohen's effect strength was calculated and graded as follows:

Cohen: d = 0.2 small effect, d = 0.5 medium effect, d = 0.8 great effect.

The correlations were determined according to Pearson, including effect size (effect force, according to Evans). Evaluation of the correlation coefficient r was graded:

< 0.2: Poor—0.2 < r < 0.4: weak—0.4 < r < 0.6: moderate—0.6 < r < 0.8: strong—r > 0.8: optimal.

The level of significance was set at p < 0.05.

## Results

A total of 13 patients (female: n = 2, 15%) with an average age of 27.6 years ± 9 (95%: 22–33 years) were included. The mean defect size was 8.3 mm ± 2 (CI 95%: 6.9–9.8 mm). Since the fracture event, an average of 21 months ± 31 (95%: 2–40 months) had elapsed before surgery. Seven patients (54%) were pre-operated with a Herbert screw (Table 1).

**Table 1:** Epidemiology of the patient group. All patients signed written ‘consent for publication’.

Pat. no	Age range	Sex	Smoker	Size of defect (mm)	Previous therapy/time since fracture
1	30–39	M	N	6	3 years, no previous therapy
2	16–19	F	N	8	Herbert screw
3	20–29	M	N	11	2 × surgery with Herbert screw
4	16–19	M	N	11	Herbert screw
5	16–19	M	N	6	Herbert screw
6	30–39	M	N	7	Herbert screw
7	16–19	M	N	7	> 6 months, no previous therapy
8	20–29	M	N	8	> 3 months, no previous therapy
9	30–39	F	N	7	> 6 months, no previous therapy
10	30–39	M	N	9	10 years, no previous therapy
11	20–29	M	Y	6	> 6 months, no previous therapy
12	40–49	M	N	8	Herbert screw
13	30–39	M	N	14	Herbert screw

Radiological examinations revealed a consolidation after 3 months in 9 patients. In the other 3 patients, the consolidation was achieved at the 6-month examination at the latest. Patients were examined 2 and 6 weeks as well as three, six, and 12 months after surgery. CT scans were done 3 months following surgery and after 6 months in case of incomplete consolidation. This results in an average consolidation time of 3.4 months. Only one patient showed a persistent pseudarthrosis after 6 months. In total, this results in a consolidation rate of 92%.

The mean scapholunate angle in the 13 patients before surgery was 59.7° ± 11 (CI 95%: 53.1–66.2°); the scapholunate angle was assessed as pathological (> 60°) in 7 (54%) patients. The surgery resulted in a reduction of the scapholunate angle to an average value of 43.9° ± 5 (CI 95%: 40.6–47.2°) in all 13 patients, which was equivalent to a baseline difference of 15.8 ± 9°. The effect size of the decrease was 1.7 (p < 0.00001). The extent of the angle reduction was trend dependent on the level of the initial value (correlation coefficient: 0.5, effect intensity, according to Evans: moderate). In all cases, a non-pathological condition could be achieved concerning the scapholunate angle (Table 2).

**Table 2:** Measurements of the scaphoid pre- and postoperative.

Pat no	Scapholunar angle preoperative	Scapholunar angle postoperative	Intrascaphoidal angle preoperative	Intrascaphoidal angle postoperative	Size of defect (mm)	Height scaphoid preoperative (mm)	Height scaphoid postoperative (mm)
1	50°	36°	60°	45°	6	23	26
2	71°	47°	72°	56°	8	18	21
3	62°	38°	57°	57°	11	24	24
4	57°	45°	41°	40°	11	23	24
5	48°	42°	49°	37°	6	23	23
6	51°	47°	66°	60°	7	21	21
7	47°	42°	63°	52°	7	20	21
8	64°	38°	60°	43°	8	25	27
9	70°	48°	60°	38°	7	19	19
10	82°	50°	63°	42°	9	24	25
11	63°	55°	59°	41°	6	23	24
12	48°	40°	60°	41°	8	22	24
13	63°	43°	56°	34°	14	19	20

Comparable results were obtained in measurements of the scaphoid humpback deformity angle (SHD-angle). Before surgery, an SHD-angle of 58.9° ± 8 (CI 95%: 54.3–63.5°) was determined on average; in 4 patients, an SHD-angle over 60° was calculated. After surgery, all patients showed an average decrease of 13.7° ± 7 to an average of 45.1° ± 8 (CI 95%: 40.0–50.1°). The difference to the initial value resulted in an effect strength d = 1.8 (p < 0.0001).

The height of the scaphoid before surgery (n = 13) averaged 21.8 mm ± 2 (CI 95%: 20.5–23.2 mm). The surgery achieved an increase to an average of 23.0 mm ± 2 (CI 95%, 21.5–24.5 mm). In 9 patients, an increase was noted, in 4 patients, the height remained unchanged. The average difference was 1.2 mm ± 1, which corresponds to an effect size d of 1.1 (p = 0.002).

When comparing the strength of the injured hand and the healthy hand, an average of 36.4 kg ± 10 (CI 95%: 30.4–42.4 kg) and 42.4 kg ± 9 (CI 95%: 37.2–47.6 kg) were measured, respectively. In all 13 cases, the healthy hand showed greater strength. The difference averaged 6.0 kg ± 4, corresponding to an effect size of 1.4 (p < 0.0001).

On the visual pain scale (0–10 cm), patients before surgery specified 6.0 cm ± 2 (CI 95%: 4.7–7.3 cm). The surgery caused a decrease to an average of 2.2 cm ± 2 (CI 95%: 1.0–3.3 cm). The difference was 3.8 cm on average (d: 1.2, p < 0.0001) (Table 3).

**Table 3:** Results of the follow-up measurements.

Pat. no	Strength injured hand (kg)	Strength other hand (kg)	Strength injured to uninjured hand (%)	Return to sports (months)	Return to work (months)	VAS last control	VAS initial	Quick DASH
1	35	41	85	6	6	1	9	0
2	28	32	88	6	3	2	8	9.1
3	46	47	98	24	1.5	1	8	0
4	42	47	89	6	3	1	3	9.1
5	45	52	87	3	1.5	2	8	15.9
6	35	39	90	6	3	3	6	13.6
7	38	40	95	5	1.5	2	4	6.8
8	34	42	81	No previous sports	3	2	6	2.3
9	25	29	86	Not possible	6	2	5	13.6
10	47	55	85	6	3	1	4	4.5
11	52	56	93	3	1.5	1	3	2.3
12	28	35	80	No previous sports	6	2	6	13.6
13	18	36	50	Not possible	6	8	8	40.9

All subjects, in which a union was achieved, already showed a subjective decrease in pain levels at the start of remobilization. In contrast, a pain reduction was not achieved in the one case of the persisting nonunion when compared to the preoperative levels.

Twelve patients reported a significant improvement; one patient showed no difference from the baseline. The evaluation of the Quick-DASH scores after the operation is shown in Table 3. The median value is 9 (Q1: 2.3–Q3: 13.6). The scores correlated with the decrease in VAS values (correlation coefficient: 0.5, p = 0.05).

All patients (n = 13) resumed work after a median duration of 3 months (1.5–6 months) post-operative. Of the 11 patients who performed sport on a regular basis before the fracture, 9 (82%) started exercising again at an average of 6 months after the last surgery.

### Complications

No surgical site infection, no impaired wound healing and no implant revision or explanation were observed in 12 of the 13 patients.

**Figure 3:**
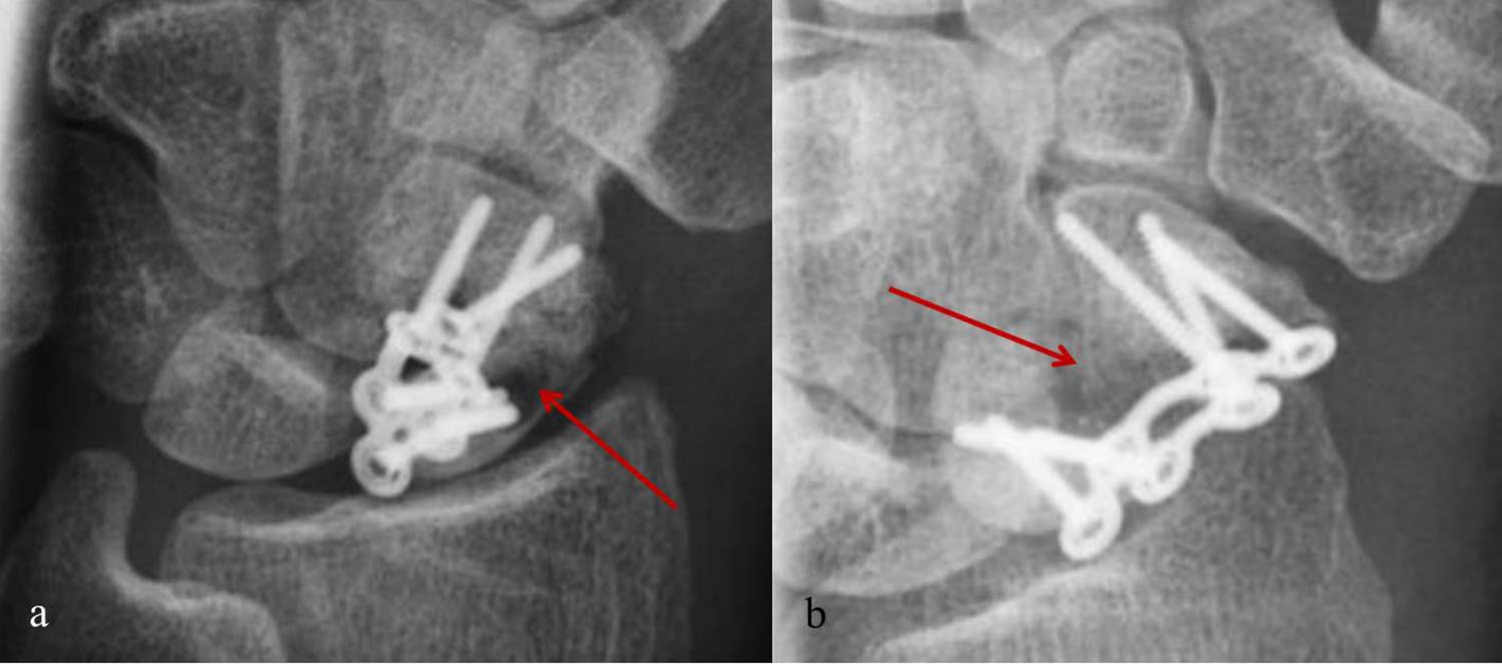
X-ray anteroposterior (**a**) and oblique (**b**). Pseudarthrosis in the Scaphoid (arrow) with retained anatomic reposition.

One of the 13 patients presented a persisting nonunion after 12 months. Initially, he displayed a sizeable intraosseous defect zone (14 mm) after screw osteosynthesis of the scaphoid. In comparison to the other subjects, the patient reported persisting pain after mobilization and a delayed return to work after 6 months. The X-ray follow-up and a CT scan after 6 months showed no consolidation of the fracture. Since the patient did not consent to an early revision operation, we kept him under regular radiological follow-up. Even after 12 months and full weight-bearing the locking plate did not show any sign of loosening or bending. The proximal and distal fragment remained in their postoperative position, and no shortening of the scaphoid or DISI misalignment was observed (Fig. 3).

After exclusion of necrosis of the proximal scaphoid fragment using magnetic resonance imaging, revision surgery was eventually performed after 12 months in the patient with persisting nonunion. Via an isolated dorsal approach, the pseudarthrosis and sclerotic zone were resected. The osseous defect was augmented by vascular bone grafting after Zaidemberg^24^. The graft was secured with a spongiosa screw and additional cancellous bone from the distal radius was filled in. The previously implanted volar locking plate was left in place and used to maintain retention during the final procedure and until healing was complete. In the course of the follow-up, a clinically good result with apparent pain reduction was observed.

## Discussion

In patients with scaphoid nonunion and severe bone defects, a treatment with a volar locking plate and cancellous bone grafting was performed. Although primary healing was not achieved in all patients, the 92% consolidation rate obtained is consistent with conventional primary treatment of scaphoid nonunions, ranging 78–92%^6^.

This shows that the advantages of the locking plate, specifically its stability and excellent retention, provide the environment necessary for osseous consolidation. In case of a large cortical defect, a loss of the reduction can occur due to the lack of rotational stability of a single Herbert screw^25^ leading to progressive loosening. In other cases, one might not have sufficient abutment in one of the fragments due to bone loss, and thus this could lead to a secondary DISI misalignment of the lunate. In our patient group, the reduction, once obtained, was retained by the locking-plate mechanism in all patients. This was even the case in the patient with persisting nonunion. Instead of the scaphoid collapsing in the developing defect zone, the plate retained the anatomy of the proximal and distal fragment even after 12 months of weight-bearing and full mobilization. Eventually, a fatigue fracture of the plate would have surely developed so that a revision was emphasized. However, we had no complications associated with the implant within the constraints of the study.

As the consolidation rates correlate with the results achieved in previous studies using a vascularized bone graft^6^, in our opinion, in the case of preserved perfusion of the proximal fragment, the use of a vascularized bone graft is not necessary as long as a stable situation is achieved. Nevertheless, a volar buttress plate osteosynthesis combined with a vascularized bone graft as already shown by Dodds and Halim 2016 as an additional procedure presents an excellent method with equal consolidation rate^17^. The duration of operation, however, can be shortened by applying an isolated cancellous bone grafting when combining with a locking plate mechanism, as in our study. This can lead not only to satisfactory union rates, but also reduce the risk of infection and anaesthesiologic complications. However, in cases of limited vascularity, confirmed by both MRI and intraoperative findings^20^, it is considered that cancellous bone grafting alone in combination with locking plate osteosynthesis may not be sufficient. In these patients, the use of a vascularized bone graft should be discussed. A further advantage of the isolated cancellous bone grafting combined with a locking plate instead of a vascularized graft is the possibility of an early mobilization of the hand. However, a comparative study on the outcome has not yet been conducted and should be evaluated in further studies.

## Limitations

This study is limited by the study design (case series) and the lack of a control group, such as pedicled graft. Also, selection bias due to only one smoker cannot be excluded.

## Conclusion

In light of the excellent consolidation rate, we regard the utilization of the volar locking plate as an essential tool in further treatment of complicated scaphoid nonunions. It should be considered in case of large and extensive bone defects. The addition of a vascularized bone graft is possible, though a cancellous bone grafting combined with an angular locking plate is, in our opinion, sufficient in many cases and leads to at least comparable results regarding consolidation and outcome. However, these conclusions may be too strong for such a retrospective study without control, further prospective studies are required to confirm these findings.
